# ‘Pearls’ of the nineteenth-century: from therapeutic actors to global commodities medicinal leeches in the Ottoman Empire

**DOI:** 10.1017/mdh.2023.17

**Published:** 2023-04

**Authors:** Büşra Arabacı

**Affiliations:** PhD student, Department of History, Hacettepe University, Beytepe-Çankaya, 06100 Ankara, Turkey

**Keywords:** Medicinal leech, Ottoman Empire, Tax-farming, Treaty of Balta Liman (1838), Global trade, Nineteenth century

## Abstract

Nineteenth-century physicians increasingly favoured leeching – the placing of a live leech onto a patient’s skin to stimulate or limit blood flow – as a cure for numerous ailments. As conviction in their therapeutic properties spread, leech therapy dominated European medicine; France imported over fifty million leeches in one year. Demand soon outpaced supply, spawning a lucrative global trade. Over-collection and farming eventually destroyed leech habitats, wreaked environmental havoc and forced European merchants to seek new supply sources. Vast colonies of leeches were found to inhabit the immense wetlands of the Ottoman Empire, which soon became a major exporter of medicinal leeches. Following the Treaty of Balta Liman (1838), the Ottoman state moved to exert control over the lucrative trade, imposing a tax on leech gathering and contracting with tax-farmers (*mültezim*) to collect the taxes. British diplomats, merchants and other stakeholders protested the imposition of the tax, as had previously happened with the commodification of wildlife; their pursuit of profit led collectors and farmers to over-gather leeches, with catastrophic consequences. By the end of the century, so great had their worth climbed that the leech population faced extinction. This paper situates medicinal leeches as therapeutic actors of history and adopts an interscale approach in formulating the human-leech interaction. It offers a substantive contribution to the history of medicine, in revealing the centrality of leeches to the rise of modern medicine and global trade, but also by making visible their role in shaping imperial diplomacy and worldwide economic markets.

## Introduction

In 2021, customs officials at Istanbul International Airport detained a male passenger carrying a suitcase containing four thousand illegally imported leeches (*Hirudo verbana*), once one of the common medicinal leech species in Turkey. On investigation, it emerged that the passenger had not obtained the requisite permit to allow him to legally transport the leeches, and he was charged with unlawfully attempting to import a regulated species. For his efforts to transport contraband items, the smuggler received a fine of 54 555 Turkish liras^1^ for breaching the Convention on International Trade in Endangered Species of Wild Fauna and Flora (CITES),^2^ under which leeches are designated protected species. Indeed, medicinal leeches were among one of the first animal species to be brought under protective conservation measures – including exportation restrictions – dating back to 1840. Around the world today, leeches are still used in several areas of modern medicine – plastic surgery, in hospital burns units – but their commercial export and import are strictly regulated, as happened after over-collecting put pressure on their supplies in nineteenth-century medicine, leading to near disappearance.

**Table 1.** A leech-centred timeline

Year	Event
Early 1800s	The therapeutic application of leeches is advertised in the newspapers.
1830s	‘Bleeding craze’ hit the rates in Europe, leech prices increase.
1830s	Early examples of farming out the leech ponds in the Ottoman Empire.
1839	The Treaty of Balta Liman is put into practice.
1840	Value of leech-inhabiting regions is assessed, and profits of leech-gathering taxes are transferred to the Ministry of Trade. Gathering leeches in bulks is prohibited.
1846	The Ministry of Finance demands a report on the operation of leech ponds from the governors.
1848	Authorities issue a warning about the extinction of leech species. The gathering and selling of extremely small and large leeches is prohibited.
1849	Mr Richard Boulth, a British merchant, protests the leech trade restrictions.
1849	Mr Eduard Rusuvich presents his report on contraband prevention.
1851	Four stores are opened in the Empire to sell the leeches at fixed prices. The tax-farmers are warned not to gather on particular sizes of leeches and not to disturb the mud of leech habitats.
1852-3	The tax-farmers are warned about the risk of extinction in reference to Mr Rusuvich’s report.
1860s	As leeches become scarce, tax-farmers seek debt relief.

**Table 2.** Leech export rates in the Ottoman Empire, presented to the Ministry of Foreign Affairs in 1852 by Mr Eduard Rusuvich

Port of export	Amount (in *ḳıyye*)	Amount (approx. in kg)^a^
Beirut (Syria)	4 500	5 769
Tarsus (Mara, Adana)	2 000	2 564
Smyrna	12 000	15 384
Dardanelles (Bergama)	600	770
Gemlik (Bursa, Bandırma)	1 500	1 923
İzmit (Bolu)	300	385
Samsun, Trabzon, Batum	10 500	13 461
Burgaz, Varna, Toulera	1 500	1 923
Silistre, Rusçuk, Nicopolis	3 500 or even more	4 487
Vidin, Harsova (Haçova)	5 000	6 410
Gallipoli (Adrianople)	3 000	3 850
Salonica (Serres, Bittolia)	7 000	8 974
Preveza (Golfe d’arta) of Janina	3 000 or even more	3 850
Valona	2 000	2 564
Durakko	3 000	3 850
Boyana, Skodra	1 500	1 923
Sum	60 900 (and possibly more)	78 087

a A *ḳıyye* is accepted as 1.282 grams. Number of leeches in a kilogram ranges from 2 500 to 200 depending on the weight of a leech, which is approximately 0.4 g to 3.4 g. Source: J.M. Elliott, ‘Population, Size, Weight Distribution and Food in a Persistent Population of the Rare Medicinal Leech, *Hirudo medicinalis*’, *Freshwater Biology*, 53, 8 (2008): 1502–1512.

**Table 3.** Medicinal leech prices in different countries in the nineteenth century

Year(s)	Country of origin	Price	Value in British currency^a^	Source
1803–1815	France	7 pounds	7 pounds	Sawyer, ‘History of the Leech Trade in Ireland’, no p. 426.
After 1815	France	15 shillings	15 shillings	Sawyer, ‘History of the Leech Trade in Ireland’, no p. 426
1830–1850	British Empire	6 pence to a shilling	6 pence to a shilling	BNA, ‘English Leeches’, *Kendal Mercury*, 6 November 1852
1830–1840	Wallachia	25 *para* to 40 *para*	~2 pence	Ardeleanu, ‘The Danubian Leech Trade’, no p. 187.
1840	Ottoman Empire	50 *gurush* preying tax	~10 shillings	BOA, I.DH. 7/340, 26 Dhu al-Hijjah [1 March 1840].
1851	Ottoman Empire	12 *para* – May to September	Less than a pence	BOA, I.MVL 214/7082, 1 Ramadan 1267 [30 June 1851].
1851	Ottoman Empire	20 *para* – September to May	~1 pence	BOA, I.MVL 214/7082, 1 Ramadan 1267 [30 June 1851].

a Exchange rates are calculated based on the values in 1844, Şevket Pamuk, *A Monetary History of the Ottoman Empire* (Cambridge: Cambridge University Press, 2000), 191.

1 pound = 110 *gurush*

2 shillings = 11 *gurush*

**Table 4.** Real and estimated values of profit of the treasury calculated by Mr Rusuvich, BOA, HR.TO 417/19, 21 Jumada al-Awwal 1268 [13 March 1852] and BOA, C.ML. 91/4109, 5 Safar 1269 [18 November 1852].

Year	Amount of gathered leeches (in *ḳıyye* )	Amount of exported leeches	Consumed within the Empire	Price per *ḳıyye*	Profit of the treasury (annually)
Pre-1852	20 000 to 25 000 *ḳıyye*s (~25 640 to 32 050 kg)	12 000 to 15 000 *ḳıyye*s (~15 384 to 19 230 kg)	8 000 to 10 000 *ḳıyye*s (~10 256 to 12 820)	400 *gurush* (~4 pounds)	20 000 000 *gurush* gathering tax (~181 818 pounds) Except the expenses 1 355 000 *gurush* was left to treasury (~12 318 pounds)
After 1852 (estimated value)	-	50 000 *ḳıyye*s or even more (~60 000 kg)	-	-	2 400 000 (24 *yük gurush*) (~21 818 pounds)

For centuries, medicinal leeches were used within folk medicine as a curative, but it was not until the nineteenth century that European physicians became almost wholly convinced of their medical efficacy. Encouraged by positive reports attesting to their effectiveness in medical cases where bloodletting was held essential for curing patients and learning from practitioners of the blood clotting properties of the anticoagulants (*hirundin*) contained in their saliva, nineteenth-century physicians throughout Europe adopted the ancient practice of venesection or leeching. Known to have originated in the healing practices of Egyptians, leeching – applying a live leech to an area of the skin to encourage or limit blood flow – was a commonplace remedy for numerous disorders and diseases, including the plague, smallpox, hypertension, epilepsy and gout, and for maintaining general health.

Medical knowledge – and popular belief – in the curative properties of these aquatic creatures spread, and across Europe, leech therapy became an established practice in medicine; in 1832, France imported more than fifty million medicinal leeches.^3^ Leech therapy was commonplace and, besides hospitals and clinics, was used in barbershops (where, in addition to shaving and hair and beard trimming, barber-chirurgeons used them in tooth extractions, amputations, cupping and other surgical operations) and by other commercial establishments touting bloodletting as a general therapy for promoting good health. Indeed, so widespread was the vogue for medicinal leeches that demand soon outpaced supply and set in motion the beginning of a lucrative global market. The leech trade was a profitable enterprise that generated huge revenues. From gatherers to merchants, those involved in the trade generated substantial profits for the Ottoman and British Empires; even the workers who gathered this prized commodity were nicknamed ‘pearl divers’.^4^

The Ottoman Empire’s vast tracts of wetlands, the natural habitats of medicinal leeches, helped to gird the foundations of the Empire’s ambitions for economic development and rise in global status, as the Empire became a major exporter of medicinal leeches to Britain, continental Europe and the United States. Following the 1838 Treaty of Balta Liman, which regulated customs tariffs for free trade between the Ottoman and British Empires, leeches became the foremost recurrent item on the agendas of consulates of foreign countries, including the Ministry for Foreign Affairs and the Sublime Porte (*Bāb-ı Ālī*), which was the central government of the Ottoman Empire. Within the Sublime Porte, a leech committee was even established with a mandate to address problems relating to the leech trade. Years after the signing of the treaty, leeches became commodified actors enmeshed within a tangle of diplomatic crises between the Ottoman and British Empires. The Ottoman state attempted to wipe out the deficits in the treasury caused by the low export duties imposed as part of the agreement of the Treaty of Balta Liman by sustaining the tax-farms (*iltizām*). The traditional system of farming out the collection of taxes in a region to individuals (*mültezim*) for three years had been applied on the leech inhabiting regions. The leech revenues were collected as a gathering tax by tax-farmers (*mültezim*) in efforts to safeguard the leech population while filling the treasury’s coffers with profits of over a million *gurush.*

A historical study of leeches is critical to understanding the substantive and important, though largely forgotten, role of wildlife in the process of the Ottoman Empire’s integration into the world economy.^5^ Indeed, as it has been suggested, medicinal leeches are the unsung heroes in the history of global capitalism.^6^ Based on archival research, this paper explores the shifting historical agency of medicinal leeches within a web of interactions to link the internal dynamics of the Ottoman Empire to global trade. It asserts that following the Treaty of Balta Liman, the imperial treasure faced the risk of losing the profits from the leech trade as the export rates decreased. In order to secure the profit, unattended leech-rich areas were farmed out to those who were in charge of collecting the leech-gathering tax. Leech farms, on the other hand, strained Anglo-Ottoman relations, as it was assumed that the application was a monopoly, which violated the free trade agreement. Despite the diplomatic disputes, imperial policies and concerns for global free trade prompted Ottoman leeches to flood European markets.

The fad for leech therapies reached its zenith in the mid-nineteenth century; by the turn of the century, European physicians had begun experimenting with new medical treatments and practices, and what had seemed to be an insatiable demand for leech therapies died. As well, over-harvesting the leech habitats led to a steep decline in their population, and the once globally lucrative trade of wildlife dwindled. It is hard to ascertain the full effect of the gathering and trading of leeches between 1838 and 1870, but so severe was the devastation to the population that leeches became – and remain – classified as an endangered species.^7^ Furthermore, as new scientific voices disputed the efficiency of hirudotherapy, public confidence, combined with prices that had risen well beyond the reach of ordinary people, saw a decline in the popularity of leech therapy.

To understand the crucial status of leeches in history, I follow an interscale approach by focusing on the human-leech interaction in three layers: individual, imperial and global. These interactions start with the leeches as the actors of medical procedures and end with their commodification in a capitalist global trade. The first section represents a historical contextualisation of medicinal leeches with their prominent role in medicine and their interaction with humans. As the healing power of the species became popular in the nineteenth-century, imperial authorities sought to secure the imperial profit by taking certain steps. Therefore, in the second layer, their role in the imperial economy – which we could refer to as leech-empire interaction – is discussed in the context of system tax-farming. To demonstrate the bureaucrats’ motivation to lessen any possible reduction caused by low export rates, this section summarises how the leech-gathering taxes were farmed out. The Treaty of Balta Liman and the circumstances surrounding the treaty’s preparation provide us with an opportunity to comprehend leech-centred diplomacy, as it is debated in the following section. The gain of the imperial treasuries, on the other hand, posed a threat to the leech population, which was one of the species affected by the free trade policies. The final layer examines the global increase in the trade of wildlife, taking the commodification and trade of leeches as an example and the extinction of their populations throughout the Ottoman lands. The narrative inevitably transitions between the medical role of leeches, tax-farms and global trade for the purpose of contributing these topics.

## Medicinal leeches as Physicians

As far as the interaction between animals and human is concerned, the activities of both are argued to be reciprocal.^8^ However, the pay of leeches in this interaction overweighs, considering their role in medicine or their agency on treatments.^9^ Hirudotherapy, or leeching, dates back to at least 1500 BC; references to the practice have been found in Jewish and Islamic books and religious manuscripts.^10^ Bloodletting was a technique used in ancient Greco-Roman medicine, beginning with Hippocrates (460–370 BC), who proposed the theory of harmony in body humours.^11^ Galen (130–201 AD) further developed the theory of humourism by linking the humours to eating habits and climate conditions. Since blood was the dominant body fluid and was believed to contain and transport the other three humours around the body, Galen suggested that it was the most important of all the humours. Venesection, or the letting of blood, was believed essential to allow excessive amounts to be discharged from the body and thus maintain the body’s harmony. Hirudotherapy was a relatively mild treatment among bloodletting remedies, such as lancing, cupping or the discharge of blood using various instruments.^12^ In *Unani* (Greco-Arab) medicine, leeches were generally used in preference to other therapeutic methods. The most detailed writing on leeching can be found in Ibn Sina (Avicenna)’s eleventh-century narration, which theorised that the removal of ‘morbid humours’ would heal the patient. Ibn Sina’s work was also responsible for popularising hirudotherapy.^13^ We also encounter accounts of leech therapy in medical books, such as Sabuncuoğlu Şerefeddin’s famous fifteenth-century manuscript, *Cerrāhiyyetü’l-Hāniyye* [The Imperial Surgery], which includes an illustration of the application process.^14^ Over centuries, medicinal leeches have been used for the treatment of eye diseases, malaria, typhoid, obesity and many other complications.^15^ Even plague-stricken patients were treated with hirudotherapy to rid their bodies of ‘harmful blood’.^16^ Leeches were generally employed for their blood-sucking activity on the human body, but other less straightforward therapies are mentioned, such as the use of dried leeches in a salve with vinegar and salt, which was then applied to the skin.^17^ Their medical agency required the scholars and gatherers to comprehend the living habits of leeches and their interaction with other species.

Studies reveal the existence of over 650 species of leeches worldwide. Within the geographical borders of the Ottoman Empire, *Hirudo medicinalis* was the common name given for exported leeches.^18^ The lifespan of a leech is up to ten years, and juveniles reaching maturity between six to twelve months.^19^ Leeches are directed by their sensory organs, which detect motion on the water’s surface. Their ability to sense the body temperature of mammals draws them, almost instinctively, to the blood source on which they feed.^20^ Paradoxically though, their heightened senses made leeches easy prey for gatherers in the nineteenth century; locals would dive into ponds and streams and present their naked feet as bait to hungry leeches.^21^ Hunger dictates the depth of their hiding places; leeches reside at different depths in their favoured water holes, depending not only on how hungry they feel, but on the water temperature. After a meal, they will remain tucked away under deeper waters beneath the rocks.^22^ It is generally thought that leeches tend to regulate their body temperature by resting in warmer places after they are satiated.^23^ A hungry leech will wait on the surface of the water for prey to create motion. Cows and horses who wander into the leeches’ habitats to swim or quench their thirst open themselves up to the ‘harmful effect’ of leeches and are sometimes blinded by an attack of the leeches to their eyes.^24^ As well as mammals, they supplement their diets with frogs, water lizards and fish.^25^ Frogs are a particularly important source of food for juvenile leeches, for the young cannot feed on the protective hard skin that covers mammals.^26^ In the absence of cattle, leeches represent a threat to the amphibian population, which provide leeches with a satisfactory source of blood.^27^ Should the ponds’ population of frogs be attacked and killed, the population of flies will increase, and they, in turn, will damage the crops in the region and as well, will be vectors for diseases around the water sources. Leeches, therefore, help maintain a balanced food chain, as they themselves are a food source for birds, fishes, turtles and ducks. The streams and lakes they inhabit are beneficial for the fish population, which in turn provide an important source of food for humans and some other animals, such as birds.^28^ Considering their little unacknowledged but vitally important place in the food chain, any decrease of the overall leech population represents a threat to local ecosystems and the wider environment.

The ideal temperature for leeches ranges from 20°C to 25°C; they cannot withstand extreme heat over 39°C.^29^ Their inability to endure cold weather forces them into a kind of hibernation, a ‘winter sleep’ during which time there is no sign of them on the surface of the water.^30^ In his *Treatise on the Medicinal Leech* (1816), James Rawlins Johnson considered leeches as barometers, able to predict the weather, although his contemporaries disputed this theory. Leeches are affected by climate anomalies such as dry winters, which reduce the overall volume of their water habitats. Similarly, leeches take refuge from very cold winters in the lower depths to hide, and they may disappear when heavy, strong winds or heavy rains disturb the waters but tend to rise to the surface before a thunderstorm – a convenient time for leech-gatherers.^31^ Because leeches rise to the surface between spring and fall, then resort in deeper waters for the winter, they fetch higher prices during the colder months, as well as during times of climate extremities when they are most scarce. Gatherers who made their livelihoods selling them had always to be attuned to the favoured water conditions of the leech population; leeches could not be collected and stored away for long periods of time, for freshly gathered leeches were highly preferred by hirudotherapists.

The vogue for leech therapy was made respectable by the spread of knowledge and regard for the more rigorous scientific procedures and practices of the new modern age.^32^ During the 1800s, pharmacists and physicians advertised in newspapers the therapies they offered, highlighting and lauding the special qualities of their leeches.^33^ In marketing medicinal leeches, physicians and other practitioners highlighted the awareness of public opinion as a key determinant factor in the social acceptance of hirudotherapy. Readers of British newspapers were educated in the breeding, incubation and hatching processes of medicinal leeches.^34^ These articles explained the processes of leech breeding and updated readers on the findings of recent research studies. Physicians reassured their patients of the safety of leech therapy, robustly countering the warnings by sceptics who believed leeches to be dangerous to human health. What separates the wider acceptance of leech therapy in the nineteenth century from earlier centuries was a new scientific curiosity – driven by Enlightenment emphasis on scientific enquiry as the route to human progress – about animals as a species.

In the 1830s, when hirudotherapy made a resurgence, many people were swept along in a wave of popularity for the ‘bleeding craze’; in Paris alone, the leech consumption rate reached two to three million annually.^35^ At the turn of the century, medicinal leeches became scarce in England.^36^ Considering the demand for the species, physicians used different methods to minimise the number of leeches used. To avoid complications, it was suggested that leeches be used only after four to five months post-therapy as a cleaning-up process.^37^ Putting full leeches onto ashes to make them disgorge blood from their swollen bodies was a common method enabling their reuse.^38^ A British physician in the nineteenth century proposed a similar method, though involving pouring salt or vinegar onto leeches to make them disgorge, which allowed their reuse up to fifty times. He also proposed cutting the belly of the leech to make it absorb more blood, further minimising the number of leeches used.^39^ Even an artificial leech device was invented by Charles Louis Heurteloup, inspired by the healing power of natural leeches.^40^ Since local supplies had reached the point of extinction, neither reusing the leeches nor inventing new instruments could meet the demand that ‘leech-mania’ caused.^41^ Quantities were low, but there remained some places where leeches could still be found and gathered, such as Cumberland, Carlisle and Penrith.^42^ Overall though, over-gathering and degradation of natural sources, poor drainage systems and cultivation of previously fallow land led to a major decline in leech populations. Artificial ponds became an alternative source for leech breeding, but they could not meet the high level of demand.^43^ British suppliers were eventually forced to look overseas to Continental Europe to purchase these valuable commodities in the 1800s.

From these calamities, it is clear that the leech commodity trade did not always run smoothly, or independently, being very much bound to the fortunes and episodic crises of foreign diplomacy. For instance, before the Napoleonic Wars, imported leeches from France cost seven pounds, but after the wars, prices plummeted to fifteen shillings.^44^ Still, the dramatic price cuts did little to entice customers to purchase French leeches; rumours spread of patients poisoned by them or of serious allergic reactions. Cautious Irish merchants and their customers shunned French suppliers, preferring instead German (or black) leeches.^45^

In addition to France and Germany, British merchants looked to Portugal, the United States and Morocco as sources of leeches for the British markets.^46^ Yet, no amount of imported leeches could be sufficient, so British merchants turned their attention to the Ottoman Empire. One reason for this was the low custom duties provided by the Treaty of Balta Liman, signed between British and Ottoman Empires. The process of putting out to contract the collection of taxes on leech gathering closely reflected the terms of the treaty, as well as the trade policies of the British Empire.

Leeches sourced from the wetlands of the Ottoman Empire enjoyed popularity in European countries, where they were in high demand, especially by the British Imperial State. In the years of trade, a British reporter described in explicit detail every step of the leeches’ journey from their natural habitats to the leech factory in Izmir, bringing to life for the newspaper’s readers the business of leech farming within the Ottoman Empire. As reported in *The Ulsterman*, leeches were gathered from their countryside habitats of streams and lakes by labourers, who splashed their naked legs in the water, creating surface motion which attracted the leeches’ attention. Once collected, the labourers brushed the leeches from their legs before sorting their catch by weight (*ḳıyye*).^47^ The leeches were then transported to the factory, re-weighed, grouped by size, placed into one of eighteen ponds and fattened on a diet of ox blood; as their weight tripled during the feeding process, the leech business became highly lucrative. To prepare the leeches for shipping, it was vital to prevent the water from stagnating, so the water was kneaded with clay, and the leeches were placed into tanks in small tubes before being sold for export to foreign countries or to ‘most parts of the doctored world’.^48^

The leech factory depicted by the reporter was the largest in the city, although other businesses competed on smaller scales. These establishments were convenient for wholesale supply, but there were also individuals who gathered leeches for trade. Foreign merchants either purchased their supplies from the tax-farmers and local labourers or gathered them – sometimes as smuggled contraband – from their water sources. As well as seeking to capture trade from foreign exporters, the leech industry marketed their supplies to locals, who bought leeches from herbalists or peddlers in the marketplaces. Bathhouse patrons could even relax in comfort while an attendant applied them to their bodies.^49^

The Sultan and courtiers had their own allocations of leeches; each year, 350 *ḳıyye*s of leeches were delivered to the Imperial Pharmacy (*Eczā’-ḫāne-i ‘Āmire*).^50^ In 1853, the private treasury of the Sultan (*Ḫazіne-i Ḫāṣṣa*)^51^ paid 8 984 *gurush* and nineteen *para* for palace use. So long as prices remained low, even disadvantaged people could participate in the global consumption of this commodity. When world prices escalated, however, leech therapy was a luxury that was now beyond the purchasing power of the poor.^52^ To overcome the problems stemming from erratic price fluctuations in the Empire, four stores (*dükkān*) were opened in 1851. From these establishments, leeches could be purchased at a fixed price of twelve *para* per leech from May to September if their wetland habitats gave up the greatest quantities of leeches. Since, as was earlier noted, weather conditions affected leech supplies, they were sold at twenty *para* between September and the following May. In harsh winters, when the waters froze, prices rose to twenty-five *para*.^53^ A similar process was set up in Wallachia, in today’s Romania, where authorities fixed the price at twenty-five *para* for the poor and forty *para* for ‘rich’ people.^54^ Other domestic customers for medicinal leeches were hospitals, barber-surgeons and pharmacists. While being actors of medical procedures, leeches gradually became commodities of domestic economics and foreign trade. The following section describes how the tax-farms were operated to increase the treasury’s income.

## The role of medicinal leeches in the imperial economy: tax-farms

Tax-farming and commercialisation activities were two factors re-formulating the network of state-society in the Ottoman Empire.^55^ The tax-farming system, which depended on the contracts on the local land, was a major force in the creation of a centralised bureaucracy that would provide the framework for the state reforms.^56^ Accordingly, tax collection for a specific region was farmed out by auction to an individual, a community or a local administrative body. The *mültezim* paid the price of taxes to the government in three instalments; in return, he was in charge of collecting the taxes for three years.^57^ Similar to every profitable product in the Empire, fishing and leech gathering were subject to tax.

Before 1839, tax revenues for leech gathering in different parts of the Empire were collected by tax-farmers on behalf of the Ministry of Finance. An example was in Rumelia, where, in 1837, orders were given for the rivers to be operated by a tax-farmer.^58^ Apparently, some parts of Rumelia were entrusted (*emānet*) by the government, while others were auctioned to individuals. Taxes for leech gathering in Aydın district and trade were auctioned off for twenty thousand *gurush* to individuals who either managed them or appointed a deputy.^59^ In 1840, it was decreed that lakes and rivers suitable for leech gathering would be operated under the control of the Ministry of Trade. The state was keen to contract the leech sources to tax-farmers in their efforts to secure the population, generate revenue and capitalise on the rising prices of leeches on world markets, as well as lightening the terms of the Treaty of Balta Liman.

The 1840 decree, which aimed to protect the treasury from the debts, provided two benefits for the state, namely, securing both treasury funds and adherence to the treaties. Transferring the tax revenues to the Ministry of Trade lifted taxes from 600 000 to 750 000 *gurush.* ^60^ The same year, gatherers whose dwellings surrounded leech habitats were levied fifty *gurush* of tax (*‘öşr*) for each *ḳıyye* of leeches they gathered. Those who transported leeches to port cities were required to pay tax which amounted to an internal custom duty. Furthermore, local people employed as leech gatherers were to receive a proper daily wage. The decree protected the natural sources, saving the treasury from potential shortfalls by salt, leech and fish tax-farms, and protected daily labourers from low daily payments.^61^ Within a decade of the decree’s implementation, the treasury’s profits increased to over a million *gurush.* However, this increase in profits was to have serious implications for the sustainability of the leech population.

In replying to the letters from the Ministry of Finance, the governors or local rulers noted the habitats suitable for leech gathering and declaring their earlier ownership status. These letters reveal how the leech taxes were farmed in different regions. One of the correspondences was with the *Mutassarifate* of Quds, who was asked specifically to provide details of the condition of places thought suitable for leech gathering, in order to farm those habitats out to a suitor from Istanbul for the next three years. The *Mutassarifate* replied, stating that the leeches were only rarely found in one or two sources, habitats which had never before been in the hands of a tax-farmer.^62^ The district chief (*ḳā’immaḳām*) of Akka replied to the same request, identifying three or four ponds and a stream in the locality that could be suitable sources for gathering leeches. In two of these reports, and similar to Quds, no one wanted to be a tax-farmer. Since everybody could gather leeches ‘as they wished’ in the absence of a tax-farmer, the Ministry decided to farm out those sources as soon as possible.^63^ In a similar vein, Tebnine Nahiyah responded to the demand for information, explaining that there were in fact no sources left to farm out.^64^ For the information demanded from Trablusşam, the district (*vilāyet*) majlis reported that there was a single pond where only two to three thousand leeches could be gathered. With help from the town crier, the authorities gathered the locals to identify individuals desirous of becoming a tax-farmer for the leech gathering, and an auction was subsequently organised for that purpose.^65^ A different resolution was found in Adana, where no one was willing to farm three lakes that were habitats for leeches. The treasury stepped in to operate the lakes for a while, but eventually, it was decided that it was not ‘convenient for a ministry to hold such a privilege’; the lakes were subsequently farmed by the Elder’s Majlis, who received one-tenth profit in return.^66^ These examples reveal the desire of the Ministry and authorities to control leech-gathering activities in different regions.

Many foreigners joined the business of tax-farming as well. Leech-gathering taxes for Ioannina, Avalonia, Delvin and Ohrid districts were farmed out to an Austrian captain for three years in three instalments of 24 000 *gurush* per year.^67^ Similarly, a Russian counsellor’s deputy contracted the rivers in Samsun for four years in return of 10 000 *gurush.* However, over five months, the deputy found many excuses to refuse to pay the price of the tax-farm; the amount was eventually demanded from the Russian consulate.^68^ Any sources of leeches, which had not yet been farmed out, held the status of *mîrî* land, and since the income belonged to the treasury, gathering leeches from these fields was forbidden. However, in some parts of the state, there was no one to claim the tax sources. In such cases, the auction prices were reduced, and if still no claimant could be found, they were entrusted to independent officers so that the sources were not left idle.^69^ Since the leech-gathering tax was controlled by the state, leeches gathered from some regions were provided directly to the hospitals and pharmacies throughout the state. If hospitals gathered leeches independently of the state, they had to pay a tax of twenty per cent, the same rate as the fishing tax in the 1850s.^70^ Although the state controlled the gathering process, tax-farmers and locals were driven by the profit motive; the consequence was the over-gathering of the leech population, which seriously impacted the long-term sustainability of the leech population.

Since all the arrangements related to tax-farming were designed to benefit the treasury, imperial law secured leech-gathering taxes in several ways. One method was the gathering licence, which aimed to prevent smugglers. Tax-farmers had the right to operate the lakes either by themselves or through an appointed representative. They also had the right to recruit gatherers and to give certificates to locals and foreigners in return for collecting the requisite tax.^71^ Gathering leeches without permission was not permitted, and anyone without possession of a certificate confirming their permission to gather leeches was charged with theft. Certificates permitting leech gathering included the name of the district (*sancaḳ*) or sub-district (*ḳażā*) and the name of the tax-farmer. Merchants who transported the leeches to port cities had to show their certificate of authorisation, bearing a valid stamp from the tax-farmer, the district chief (*ḳā’immaḳām*) and the administrator (*müdîr*). Without the possession of a certificate, transporters were denied passage through customs and were unable to export their loads, as transporting and trading leeches without a certificate was illegal.^72^ In such cases, the regional authority (*ḳocabaşı* or tax-farmer) were entrusted with the right to seize the leeches at the customs point.^73^ Foreign merchants who could not show a certificate were required to pay the leech gathering tax at ports before shipping.

Some merchants resisted paying the tax, arguing that it should have been demanded during the gathering process in the field rather than at the customs house. Furthermore, if it became known that the leeches had been sourced from *mîri* land, any revenues would belong to the treasury, and so they objected to paying the tax on the grounds that the site was open to the public.^74^ In instances where the source fields had not yet been farmed out to anyone, leeches gathered from here were confiscated by the state. As an example, a man from Iznik named Yorgi was caught with 120 *ḳıyye* leeches, which were seized for the benefit of the treasury. He was sentenced to prison as he ‘dared to prey on leeches’.^75^ The process discussed so far takes us to the measures that were put in place within the Empire and which obliged British merchants to pay regular leech-gathering taxes. Following the free trade agreement, tax-farms along with the monopolies became one of the hotly contested issues surrounding the leech trade.

## Disputes over leech trade following the Treaty of Balta Liman

Throughout the nineteenth century, Anglo-Ottoman relations changed direction according to the domestic and foreign affairs of the two Empires. The Greek Revolution of 1821 altered Anglo-Ottoman relations by initiating a series of conflicts that culminated in the defeat of the Ottoman navy by French and British fleets in the Battle of Navarino (1827). When the Sublime Porte demanded aid from Egyptian Governor Mehmed Ali Pasha to suppress the Greek revolution in Morea, Pasha successfully put down the revolt but became a threat to the central government. Mehmed Ali Pasha eventually marched against Sultan Mahmud II in 1833. To counter the threat he posed, the Sublime Porte signed with Russia the Treaty of Unkiar Skelessi (1833), an act that created problems for Anglo-Ottoman relations. However, it also had the effect of moderating British foreign policy against the Ottoman Empire, as the British Foreign Office (BFO) decided to support the Sublime Porte in foreign diplomacy, as well as the Ottoman Empire’s reform process.^76^ The reforms represented steps in the modernisation of the Ottoman Empire, as the Edict of Tanzimat (1839) ushered in reforms in administration and economy, and in the military institutions. However, earlier crises and wars, as well as the planned reforms, placed severe burdens on the treasury, which required the Empire to profit from tax revenues and foreign trade.^77^

While the Ottoman Empire was transitioning through its reform period, the British Empire set about improving its commercial relations with different countries by supporting the free trade policy. The idea of embracing a free trade policy can be traced back to the eighteenth century, though, after Parliament’s 1820 decision to enhance the commerce, it was believed by economists that free trade would increase the ‘real income’ of the British state.^78^ To further encourage the process, the British state entered into various treaties with several states including Austria, France, Prussia, China and Russia.^79^ To this end, the Treaty of Commerce was signed between Austria and Britain in 1839, the same year that the Treaty of Balta Liman took effect. As Lord Palmerston stated during a parliamentary sitting, the two commercial treaties ‘were likely to be attended with very great advantages to the commerce of this country.’^80^

The BFO’s policies of ‘protecting the territorial integrity of the Empire’ while they stood to gain diplomatic and economic benefits^81^ motivated them to sign the Treaty of Balta Liman. Both the issue of Egypt and the ongoing Tanzimat reforms were crowned with the treaty, which created an alliance with the British Empire that lasted through to the Crimean War. The Treaty of Balta Liman proved most favourable and profitable for the British Empire, which was in search of a new market for British goods; it also served to lessen the economic crises within the Ottoman Empire.^82^ In Lord Palmerston’s view, ‘Britain would gain by having a prosperous customer’, and the imperial coffers would also swell with domestic income accruing from the prohibition of monopolies throughout the Ottoman Empire.^83^ In the immediate period following the treaty’s signing, export rates increased due to the abolishment of the restrictions on the export of agricultural products.^84^ The effect of the treaty was to quadruple export taxes while keeping import taxes low, an outcome which increased goods in the Ottoman markets and speeded up the state’s industrialisation process.^85^ The Treaty of Balta Liman has been hotly debated in terms of its articles and the privileges it gave British merchants. Three treaty-related issues sparked tensions in Anglo-Ottoman diplomacy: conceptual debates, translation crises and British merchants’ rights.

In particular, the second article permitted the British merchants to purchase, without exception, any commodities produced, grown or manufactured in the Ottoman lands, whether for internal use or foreign trade.^86^ This became a reference point for protests against the Ottoman Empire’s tax policies by the foreign leech merchants. As well as preventing the Ottoman authorities from restricting export commodities, the same article stipulated the abolition of monopolies or *yed-i vaḥîd.* ^87^ The system of *yed-i vaḥîd* had been put into practice in 1828 to increase the treasury’s profits and restrict the exportation of some commodities by maintaining control of the state.^88^ The state monopolised a trade article with the private ownership, and it had been applied first to control opium production and trade. Accordingly, only licenced merchants having permission from the *yed-i vaḥîd* owners were allowed to sell opium.^89^ Also, in case there was scarcity of merchandise within the country, it could be embargoed from exportation, and in times of war, additional taxes would be applied to imported and exported commodities.^90^ However, the Treaty of Balta Liman abolished all restrictions on trade, including monopolies. The letter below, written on behalf of the Ministry of Trade, underlines the different understandings of the concept of the monopoly held by Ottoman and British authorities.

Since any kind of monopoly ‘was not in accordance with the articles of the treaty’, the Ministry of Trade took over taxes on leech sources in 1840. As the reason for this transformation, a ministerial letter stated that the foreign consulates had been objecting to monopolies or raising ‘unnecessary voices’, which were to be avoided considering the ‘sensitive times’ currently obtaining in the empire.^91^ Basically, any kind of conflict over monopoly was avoided by the ministry by emphasising that the decision was entirely in line with those treaties which had created the conditions for free trade (*serbest ticāret*). It was further claimed that the state was merely following a key condition of the treaty on the abolishment of monopolies (*yed-i vaḥîd* ve *inḥiṣār*) on certain goods.^92^ Following the decision, the Ministry of Trade would be able to control the leech trade as well as the tax revenues.

While the Ottoman officers conceded the incompatibility of monopolies with free trade, they studiously excluded the matter of the tax-farms from their discussions. In essence, for the Sublime Porte, the taxes were not related to customs duties, but instead, a tax was imposed on leech gathering. The fourth article of the Treaty of Balta Liman fixed the export duty at nine per cent *ad valorem*, free of any additional charges incurred in transporting the goods. However, the twelve per cent leech-gathering tax, increased the tax demands on the merchants. This amount would be paid to the tax-farmers directly. As documented in the correspondences of Sir Stratford Canning, the ‘Great Ambassador’ and second-most prominent figure in Anglo-Ottoman diplomacy after Foreign Secretary Lord Palmerston,^93^ the BFO strongly opposed tax-farms, charging that they were a part of the monopoly system. Between 1840–1850, the ambassador’s word choice for tax-farms changed from ‘monopoly’ to ‘partial monopoly’ and eventually to ‘farming out the taxes’. For Canning, the main problems of the Treaty of Balta Liman were ‘monopoly, road duties and retail trade’.^94^ Months later, he also condemned an ‘unwise system of partial monopoly’ followed by the Porte, by farming out the revenue to high-rank officers.^95^ The specified partial monopoly confused the British foreign officers so much so that a definitive report on what actually defined a monopoly and whether tax-farming should be regarded as an *appalto* was requested from the consulates.^96^ Their conflicting views on the issue would increase the diplomatic tension between the countries in the following years.

In 1849, Richard Boulth, a British merchant, was about to ship a tube of leeches from Smyrna (Izmir) to Marseilles.^97^ Customs officers forbade him from loading the tube for shipping as he did not show the requisite licence. In a letter to the consulate in Smyrna, Boulth complained about the actions of Abdülkadir Pasha, the customs officer. In response, the customs officer defended his actions denying Boulth’s right to load the leech tubes, for the merchant was unable to show a certificate proving the leeches’ provenance or confirming their legal purchase.^98^ Boulth protested, asserting his right to trade any industrial or agricultural item produced in the Ottoman lands by referring to the second article of the Treaty of Balta Liman.^99^ When another complaint protesting the leech monopoly was handed over by a foreign merchant in Salonica, the district governor rebutted that the leech trade had never been a monopoly (*yed-i vaḥîd*) but had been passed to tax-farmers for the purpose of protecting the lakes.^100^

Besides medicinal leeches, some other items (salt, alum, snuff and war materials) were exempted from the terms of the treaty on the grounds that they were bounded to the tax-farmers and could not be sold or traded without the permission of the tax-farmers.^101^ These articles were fundamental for the imperial economy and were therefore treated in a different way. The situation created discomfort among the British merchants.^102^ In the following years, British diplomats and journalists continued their criticisms of tax-farms. A journalist from the *Morning Advertiser* criticised the policies of the Porte in a letter. He railed against the ‘illegal’ and ‘exceptional’ taxes demanded by the Ottoman Empire and fumed that ‘local authorities monopolise’ every village; except for the capital, he continued, every district remained dependent on the taxes and continued the practice of monopolies.^103^

In fact, the problems of the treaty were not only relevant to the leech trade; disputes over translation constantly threatened to derail the treaty negotiations. The Sublime Porte questioned the Turkish version of the third article because it did not include a translation of the expression ‘in any manner of trade therein’ within the English version.^104^ The British Foreign Office claimed that the meaning was already inherent within the second article, which gave merchants the right to wholesale trade. The British Foreign Secretary doubted that the intention of the Porte was to relieve the obligations of the convention by not recognising the difference in translation.^105^ Since similar inconsistencies in the translation occurred in the English version, the treaty did not come into operation until 1 March 1839.^106^

The third issue raised by the treaty concerned the rights of British merchants. Under the first article, it was accepted that all the privileges and immunities provided to the ships and merchants of other countries would be provided to Britain. In 1843, British merchants objected to the British embassy over the advantages given to Russian merchants – or ‘the subjects of the most favoured nation’ – who were exempt from payment of an additional two per cent import and nine per cent commutation duties. The British embassy tried to delay the negotiations between Russia and the Ottoman Empire until they had secured the same terms for British merchants. ‘The fulfilment of the treaty stipulations in favour of Great Britain’^107^ was the main aim, but the Lords Committee saw the risk of losing all privileges granted to the British merchants on internal duties at the expense of the comparatively moderate two and nine per cent payments. Meanwhile, the committee insisted that Britain’s government had no right to demand exemptions, forcefully reminding the lords that it was the right of Ottoman bureaucrats to act as they wished. In essence, insisting on exemption at the same rate as the Russian merchants might ‘force [the British] into a worse condition’.^108^ In 1845, on the eve of negotiations between Russia and the Ottoman Empire, the news of more privileges to be granted to the Russian merchants reached the British newspapers. Reporters asserted that if Russian merchants were accorded more commercial privileges than their British counterparts, the Treaty of Balta Liman would be repealed.^109^

The conflict over the privileges granted to Russian merchants caused further disagreements between Britain and the Ottoman Empire during the renewal of the tariffs. As Mübahat Kütükoğlu states, the Sublime Porte resorted to changing the second article to prevent the export of every kind of commodity produced within the Empire.^110^ She further claims that Britain agreed to change the article only on condition of a fair agreement between Russia and the Ottoman Empire. Eventually, a commercial treaty was signed between Russia and the Ottoman Empire in 1846 at Balta Liman, the same place where the treaty with Britain had been signed. The treaty with Russia was acceptable to the demands of the British Foreign Office, but the demand of the Sublime Porte on re-sitting the second article of Balta Liman was not settled.^111^

Considering the years of negotiations, the dates of the decrees regulating leech gathering overlap them. The Corn Laws were repealed in 1846 when this period was widely perceived as marking the start of a new era of free trade.^112^ In the same year, negotiations started for the renewal of the tariffs between the sides when the Ministry of Finance had been recording convenient sources of leech gathering. Whereas the taxes had hastened the farming process, the crises over the leech trade continued. In the tariffs of 1850, the British ambassador demanded the reduction of customs duties, which was accepted with a discount of sixteen per cent on exported articles.^113^ However, it is no coincidence that between the tariffs of 1850 and 1862, the price of leeches significantly increased by 750.4 per cent.^114^ As a source of income for the treasury and profit for the trade, the actors of medical treatment had become a global commodity.

## Leeches in the global trade and their extinction

Affected by the free trade policies, medicinal leeches in the Ottoman Empire became an integral factor in global trade owing to their use in medical procedures. In 1832, France imported 57.5 million leeches from various countries. By 1852, ten million leeches were imported half of which sourced from the Ottoman Empire.^115^ The reason for the decrease of the leech importation for France was either because of fading of the treatment or the developing of new methods of leech farming. However, the increasingly competitive place of the medicinal leeches in global trade is closely related to free trade policies and the increase of the commodities on global markets.

The Ottoman Empire exported many animal products, including goatskins, sheepskins, hare skins, silk cocoons, wool, buffalo horns and ox horns, to European countries, but leeches were the most valuable.^116^ It was the opening of a new capitalist chapter for the leech trade^117^ as the motivation to kill animals ‘more than necessary’ accompanied the rise of the capitalist economy.^118^ Free trade gave locals and foreign merchants greater motivation to gather larger volumes of leeches to sell on global markets as was the case for the trade of other wildlife items in the nineteenth century. The Indian fur trade had encouraged local people to kill more animals to meet British demands.^119^ Meanwhile, in America, buffalo and bison were slaughtered for their skins in mass numbers which considerably reduced their population.^120^ Entering the world economy led to the commodification of nature and of natural products, such as ivory and furs. The integration of animal species into the global trade fuelled more demand than nature could supply and significantly decreased the populations of many of the world’s species.^121^

Technology and transportation changed trading conditions while facilitating integration into the global economy.^122^ Along with technology, industrial development played a role in easing the transport of leeches. Steam trains and steamships delivered leeches once carried on horseback to far distances.^123^ When they were carried by wagons and ships, unhygienic conditions were a danger for leeches; therefore, leeches had to be washed and sealed against contagious diseases. This explains why the British journalist visiting the factory in Izmir emphasised the hygienic conditions. Great care also had to be taken when the leeches were stowed aboard the ship; the water they were preserved in had to be changed every two days.^124^ Following transportation to ports, the leeches were kept in artificial ponds, such as in Bordeaux.^125^ Once the leech trade became a part of the trans-Atlantic trade, the majority of leech tubes were brought to Lisbon, the principal entry point for the flow of products from the Eastern Mediterranean to Europe. Following this route, leeches which were shipped from the Ottoman Empire were called ‘Green leeches’, or ‘Lisbon leeches’.^126^ Foreign companies controlled many of the trade stations or agreed with locals to cooperate on the leech sources, as happened in the Balkans. These companies monopolised the leech trade in the region, and they dominated the trade even after leech exportation was banned, but it did not stop the smugglers.^127^

The Sublime Porte knew that farming out the leech sources could cause some problems, such as over-gathering from the ponds.^128^ Tax-farms, which were supposed to control the gathering activities, ended up with the commodifying of leeches. In this period, foreign merchants objected to the state sanctions for illegal gathering. In cases where no certificate was shown, the authorities seized the leeches even as foreign merchants were ‘claiming that they have permission to take anything’ produced in the Ottoman lands and vociferously protested the state’s seizure of ‘their’ leeches. The rights of tax-farmers to confiscate leeches was conferred onto foreign consulates, but they responded that it was the duty of tax-farmers to protect the lakes under their jurisdiction. They argued that if they did not protect these valuable aquatic creatures, the merchants could themselves gather the leeches. The British consulate protested the confiscation of leeches ‘in accordance with the contract in their hands’, claiming that their merchants had the right to take and sell any goods produced in the Empire. If the state seized the leeches, they would protest the state and claim recompense for the damage caused.^129^ Sir Stratford Canning, who promised to pay special attention to the British merchants,^130^ strongly opposed the Sublime Porte’s actions, stating:

Although pools wherein leeches are found, may be farmed out to particular individuals, the exportation of their produce, that is the traffic in leeches, cannot be made an object of privilege, except on the principle of monopoly and all monopolies are expressively abolished by the Convention of Balta Liman.^131^

Two years later, he criticised the tax-farming of leeches further:

In the matter of leeches, as in that of salt, the Porte advances pretentious which are not borne out by the Treat of Balta Liman. No one disputes the right of the Porte to farm out the produce of its leech ponds. These ponds are in most instances, if not in all, the property of the State, and the right of the state to dispose of its produce cannot be left them the right of any individual to dispose of his own property (…) It is quite intolerable that, in order to save that expense, they should be allowed to seize leeches in the population of British merchants.^132^

The Sublime Porte did not want to arouse discontent, but for years, complaints continued to issue forth from consulates and tax-farmers who demanded their rights to collect taxes on leeches. The consulates of France, Britain, Germany and Italy each dispatched letters to the governor of Salonica, claiming that tax-farmers were empowered to gather leeches only in the morning. Accordingly, tax-farmers should be protecting these places during the night, and if they did not, they had no right to seize these leeches.^133^ The complaints by the foreign merchants included the claim that they had the rights to gather and sell leeches ‘anywhere they wished’.^134^ Such claims were not peculiar to the British merchants but came also from Russian, Austrian, French and Belgian merchants, underlining the global scope of the leech trade.

To tackle the crises in the leech trade, the Leech Committee (*Sülük Ḳomitesi*) was appointed by the Sublime Porte, consisting of members representing different countries. To this Commission, the consulate of France in Istanbul appointed one of the dragomans, Monsieur Dopre, as ‘this article is very precious for the trade of France’, and as they wanted to have notice of every move the Commission made.^135^ It is not clear what exactly was the nature of the Commission, but it might today be considered a supra-state organisation, for the leeches were the common matter between all the countries involved. Some decisions that were related to leech-gathering taxes were left to the Commission. For example, an American merchant who had been a tax-farmer of leech sources in Izmit demanded an exemption from his taxes as he could not make a profit. The decision was left to the findings of an investigation by the Commission, which ‘was constituted from necessary people and officers’.^136^ Considering the sensitivity of the situation and the desire to have a share in the merchandise, it is not hard to understand why the consulates wanted membership of it, as in the case of the appointment of Monsieur Dopre.

To reduce costs and meet the demands, *Hirudiniculture,* or leech farming, became a preferred form of entrepreneurship in most countries. In Brazil in the 1830s, leech farms were built to increase the leech population.^137^ Building artificial sources was one way of tackling the ever-decreasing natural sources, but the problem of near extinction was now on the agenda of many countries. To overcome the problem of population decline, in 1859, the Portuguese Royal Academy of Sciences offered a prize to anyone who could find the best artificial condition within which to breed leeches.^138^ In Wallachia, prices rose to a point where the leech became a ‘luxury product’ in the markets. To protect the population, authorities outlawed leech export from 1835 until 1847, after which time exports were limited to a set number per year.^139^ These efforts to preserve leech populations were made years before comparable attempts by the Ottoman Empire, which decreed restrictions on gathering and exporting leeches much later.

Amongst all the protests, one merchant struggled to protect the leeches. In Shkodra, Eduardo Rusuvich objected to tax-farmers who helped the illegal gathering of leeches on *mîrî* lands. Rusuvich came across some merchants not in possession of the requisite certificates and guessed that they were illegally gathering leeches from the region. He warned the authorities many times to take action to prevent illegal leech gathering because it was ruining the region’s marshes and depleting the leech population. He sent his protest through the British consulate in the region, explaining that he felt it was ‘his debt to pay to prevent this’ contraband trade.^140^ Rusuvich presented a report to the Ministry of Foreign Affairs estimating the revenue to be made by stamping out illegal leech gathering and urging the more robust control of gathering activities in order to benefit from the extra income taxes.^141^ He also proposed that leech merchants who damaged the leech population and the environment should be denied certificates and recommended that action be taken to prevent tensions between merchants and the state, for such disagreements led to leeches being left to die at the ports and other places they had been seized.^142^ Even before Rusuvich’s warning, since 1840, it had been made illegal to gather copious quantities of leeches. His report represented one individual’s efforts to raise awareness of the potential loss of revenue due to poor administration of the leech gathering trade and the likely destruction of the leech population.

In 1848, customs officers received warnings of the economic and environmental dangers posed by the extinction of leeches. Bans were imposed on the gathering and transporting of very small or large leeches. Customs officers who found merchants carrying leeches of the prescribed size were empowered to seize these shipments on behalf of the treasury.^143^ These rules were reiterated to gatherers in 1851, along with additional warnings that they avoid damaging the leeches’ muddy habitats during the gathering process.^144^ These measures were taken to protect both the treasury and the leech population. In 1853, a debate was held on limiting the harvesting months, thus ‘protecting leeches from any damage’.^145^ Dire warnings were sounded of a bleak future should everybody be permitted to gather leeches without restriction; within a couple of years, ‘not a single leech will be found’.^146^ The warning was realised in the 1870s when leech and other tax-farms in many districts went out of business.^147^ The debts that accrued from unpaid taxes were inevitable. Mehmed Beg of Aksaray owed 15 900 *gurush* to the treasury,^148^ and the tax-farmer of Kütahya owed 12 000 *gurush* worth of leech tax in 1856.^149^ In these years, the dramatic decline of the leech population became more apparent when tax-farmers could not collect sufficient leeches to meet the tax demands. Bekir Aga, a tax-farmer from the Kayseri district, successfully requested forgiveness of his debt of 18 500 *gurush* to the treasury, for he made no profit and had not been able to collect sufficient tax.^150^ The same request was made by the tax-farmer of Konya district, who was unable to pay three years’ worth of taxes of 63 000 *gurush.* A field investigation discovered that leeches existed only in some parts of the district and, moreover, only in small amounts.^151^ Many other tax-farmers requested debt forgiveness, while yet others were seriously affected by the regional scarcity of leeches. It is possible that the breeding and reproduction of the leech population was affected by the extreme heat and drought of the second half of the century. Animals suffered terribly at such times, which brought starvation and gave rise to diseases.^152^

The eventual near extinction of leech populations brought the loss of huge revenues to the treasury. For nearly two decades, the leech trade was a significant driver of the imperial economy, a status it held until leech therapies fell out of favour in the last quarter of the nineteenth century. New studies critiqued the effectiveness of hirudotherapy; various scholars such as Pierre Louis (1787–1872) and his contemporaries conducted studies that undermined claims to the efficacy of bloodletting.^153^ Studies by Pasteur, Koch and other proponents of the *germ theory of disease* revolutionised medicine through their experiments.^154^ Belief in the healing promises of hirudotherapy declined rapidly in the latter half of the nineteenth century, and consequently, the global trade in leeches plummeted.

It is hard to measure the extent of the adverse effect of the scarcity of leeches on the environment during the course of the nineteenth century. Leech therapy’s renaissance occurred in the last half of the twentieth century, giving rise to ‘a new era of leech mania’ after the 1970s when scientists of the post-World War II era renewed investigations into their potential role as an anti-inflammatory and blood coagulant in modern health care.^155^ Today, leech therapy has regained a substantive place in human and animal medicine and as raw material in the billion-dollar cosmetics and pharmaceutical industries. International organisations such as the *International Community of Hirudopractitioners* and the *International Leech Organisation* endorse hirudotherapy and leech breeding, suggesting its growing respectability within mainstream healthcare.^156^ However, their global protection did not come until the 1989 Act of Trade in Endangered Species, which listed medicinal leeches among species to be limited within international trade.^157^ Accordingly, over the following years, *Hirudo medicinalis* and *Hirudo verbana* were subject to export limitations from Turkey. To reflect on the centuries-long journey of a commonplace aquatic sea creature, to its transformation into one of the world’s most highly prized commodities, to its eventual modern day status as endangered species is a truly sobering lesson about the destructiveness of interactions between animals and humans.

## Conclusion

Medicinal leeches have been actors in the healing and treatment of many diseases over the centuries. Their role in medicine made them valuable in their interaction with people in the nineteenth-century world. It is possible to follow transformations in medicine, consumer culture, diplomacy and trade through the historical role of leeches. Most importantly, the interplay of many animal species and their complex relationships with and to humanity have also changed over the years. This paper has shown how medicinal leeches became a global commodity, enmeshed in layers of complex interactions with humans because of their acclaimed medicinal healing powers. Leeches performed critical functions in maintaining the ecosystem and were integral to the development of modern medicine and the world economy. While leeching was a common practice, the importance of leeches in medicine brought the species face to face with empire-wide policies of tax-farming and free trade. Reforms of the tax-farming system secured taxation from leech trade for the Ottoman Empire but created diplomatic discord between two major empires of the day. In essence, the tax-farms represented the Ottoman Empire’s shrewd navigation of the restrictive elements of the articles of the Treaty of Balta Liman, which favoured British trade interests.

Towards the turn of the century, changing currents in science and the rise of the *germ theory of disease* suggested that diseases were caused by the presence and spread of germs and underlined the importance of hygiene; the outcome was the fall from public favour of leech therapies. In the same period, medicinal leeches faced the danger of extinction. Although individuals such as Rusuvich and empire-wide policies strove to protect their population, the commodification of these aquatic creatures as a prized product in the global trade imperilled their species’ existence. It may be interpreted that attempts by the Ottoman state to protect the treasury also represented, by extension, a model of a state’s efforts to protect and make sustainable its natural environment. Today, protecting the global population of medicinal leeches remains a concern on environmental agendas, though it also remains imperative to raise awareness of their ecological and medicinal importance at local, territorial and global levels.

## Footnotes

1 This amount was roughly £3 700 in 2021, and the price of a single leech is around £5.00 as of 2022.

2 ‘İstanbul Havalimanı’nda 4 bin canlı tıbbi sülük ele geçirildi’, *Demirören Haber Ajansı*, 25 December 2021, reported by Enver Alas, https://www.dha.com.tr/ (accessed 6 January 2022).

3

Constantin Ardeleanu , ‘The Danubian Leech Trade in the 19^th^ Century: the Global Market of a Tiny Product’, Revista istorică, 29, 1–2 (2018), 179. Also, for a chronological overview of leech-centred events, see Table 1.

4 BNA (British Newspaper Archives), ‘A Chapter About the Leeches’, *Bell’s Weekly Messenger*, 19 January 1852, 6. As it was so valuable and common in Anatolia and Rumelia, there were some districts named after it, such as *Sülük Divanı* or *Bingazi Sülük Nahiyesi.*

5

Onur İnal , ‘One Humped History: the Camel as Historical Actor in the Late Ottoman Empire’, International Journal of Middle East Studies, 53, 1 (February 2021), 2 .

6 Sergey Saluschev, ‘Of Leeches and Men: the South Caucasus in the Global Trade in Medicinal Leeches in the Nineteenth Century’, *The Jordan Center Blog*, NYU Jordan Center for the Advanced Study of Russia (January 2022), (https://jordanrussiacenter.org/news/of-leeches-and-men-the-south-caucasus-in-the-global-trade-in-medicinal-leeches-in-the-nineteenth-century/#.YyG_UC_OnBI), (accessed 10 September 2022).

7 In 2021, exportation of *Hirudo verbana* is restricted to 2 000 kg. *Resmi Gazete*, https://www.resmigazete.gov.tr/eskiler/2020/11/20201128-12.htm, (accessed 6 January 2022).

8 In Old English, *Læce* referred to both physician and leech. Robert N. Mory et al., ‘The Leech and the Physician: biology, Etymology, and Medical Practice With *Hirudinea Medicinalis*’, World Journal of Surgery 24, (2000), 878 1083325910.1007/s002680010141.

9

Wiebke Reinert , ‘Enlivening Exhibitions: zoos, Open-air Museums, and the History of Living Animals in Human Sceneries of Display’, Ethnologia Europaea, 49, 2 (2019), 18 .

10

Abdualrahman Abdualkader et al., ‘Leech Therapeutic Applications’, Indian Journal of Pharmaceutical Sciences, 75, 2 (2013), 128 PMC375784924019559.

11 Iain Whitaker *et al.*, ‘*Hirudo medicinalis*: ancient Origins of, and Trends in the Use of Medicinal Leeches Throughout History’, *British Journal of Oral and Maxillofacial Surgery,* 42 (2004), 133–137.

12

Lois N. Magner , A History of Medicine (London/New York: Taylor & Francis, 2005), 125–129 .

13

Azad Hussain Lone et al., ‘Leech Therapy-a Holistic Approach of Treatment in Unani (Greeko-Arab) Medicine’, Ancient Science of Life, 31, 1 (2011), 31–3522736888PMC3377041.

14
*Cerrāhiyyetü’l-Hāniyye*, folio 66, Paris Bibliothéque Nationale. Suppl., Turcs, 693.

15

Roy T. Sawyer , ‘History of the Leech Trade in Ireland, 1750-1915: microcosm of a Global Commodity’, Medical History 57 (July 2013), 421 10.1017/mdh.2013.21PMC386594324069887.

16 Tabîb İbn-i Şerîf, *Yâdigâr: 15. Yüzyıl Türkçe Tıp Kitabı Yâdigâr-ı İbn-i Şerîf* (Istanbul: Merkezefendi Geleneksel Tıp Derneği, 2017), 310.

17
*Ibid.*, 341.

18 However, recent studies have emphasised *Hirudo verbana* and *Hirudo sulukii*, two other species found in the wetlands of modern Turkey that were exploited for medicinal use. Naim Sağlam *et al.*, ‘A New Species of *Hirudo* (Annelida: Hirudinidae): historical Biogeography of Eurasian Medicinal Leeches’, *Biomed Central Zoology*, 5 (2016), 1–5; Naim Sağlam, ‘Bazı Tıbbi Sülük Türlerinin (*Hirudo medicinalis* L., 1758 and *Hirudo verbana* Carena, 1820), İhracatı, Korunması ve Sürdürülebilirliği’, *Journal of Fisheries Sciences,* 5, 1 (2011), 1–15.

19

Mahesh Kumar and Rani Manju , ‘Leech Farming in Natural Habitat: an Observational Report’, AYUHOM, 7 (2020), 17 .

20

J. Malcolm Elliott and Ulrich Kutschera , ‘Medicinal Leeches: Historical Use, Ecology, Genetics and Conservation’, Freshwater Reviews, 4, 1 (2011), 29 .

21 BNA, ‘Half a Dozen Leeches’, *The Ulsterman*, 25 January 1854.

22

Michael H. Dickinson and Charles M. Lent , ‘Feeding Behaviour of the Medicinal Leech’, Journal of Comparative Physiology, 154, (1984), 455 .

23

Ann Petersen et al., ‘Leeches Run Cold, Then Hot’, Biology Letters, 7, 6 (2011), 941–943 2155122310.1098/rsbl.2011.0320PMC3210650.

24

Mukesh C. Bhatnagar and Geeta Bansal , Krishna’s Non-Chordata: invertebrate Zoology (Delhi: Krishna Prakashan Media, 2014), 3264 .

25

James Rawlins Johnson , Treatise on the Medicinal Leech (London: Longman, Hurst, Rees, Orme, and Brown, 1816), 61 .

26 Elliott and Kutschera, *op. cit.* (note 20), 27.

27

Juha Merila and Mattias Sterner , ‘Medicinal Leeches (*Hirudo medicinalis*) Attacking and Killing Adult Amphibians’, Annales Zoologici Fennici, 39 (2002), 343 .

28 Bhatnagar and Bansal, *op. cit.* (note 24), 3264.

29 Elliott and Kutschera, *op. cit.* (note 20), 29.

30 Sağlam, ‘Bazı Tıbbi Sülük Türleri’, *op. cit.* (note 18), 5.

31 Johnson, *op. cit.* (note 25), 45.

32
*Ibid.*,18.

33 Sawyer, *op. cit.* (note 15), 423; Roy T. Sawyer, ‘The Portuguese Leech Trade in the 19th Century: the First Trans-Atlantic Commerce in Medicinal Leeches’, *Anuario do Centro de Estudos de Historia do Atlantico,* 7 (2015), 290.

34 BNA, ‘Medical Leech and Its Reproduction’, *The Atlas*, 7 October 1854, 737.

35

Roderick E. McGrew , ‘Bloodletting’, *Encyclopaedia of Medical History* (London: Macmillan Press, 1985), 33–34 .

36 Sawyer, ‘The Portuguese Leech Trade’, *op. cit.* (note 33), 295.

37

Heinrich Stern , Theory and Practice of Bloodletting (New York: Rebman Company, 1915), 82 .

38

Ahmed Yüksel Özemre , Üsküdar’da Bir Attar Dükkânı (Istanbul: Kubbealtı, 2007), 19 .

39

Codell Carter , ‘Leechcraft in Nineteenth Century British Medicine’, Journal of the Royal Society of Medicine, 94, 1 (2001), 4010.1177/014107680109400114PMC128007311220072.

40 Stern, *op. cit.* (note 37), 80.

41 Magner, *op. cit.* (note 12), 254.

42 ‘Extraordinary value’ of leeches were collected from West Water and Irish Sea, BNA, ‘English Leeches’, *Kendal Mercury*, 6 November 1852. Also, see Table 3 for varying leech prices by country.

43 Sawyer, ‘The Portuguese Leech Trade’, *op. cit.* (note 33), 295; Johnson, *op. cit.* (note 25), 41.

44 Seven pounds were equal to the price of a cow in the 1810s, or 46-days salary of a tradesman, *National Archives* Currency Converter, https://www.nationalarchives.gov.uk (accessed 6 January 2022).

45 Sawyer, ‘History of the Leech Trade in Ireland’, *op. cit.* (note 15), 426.

46 Sawyer, ‘The Portuguese Leech Trade’, *op. cit.* (note 33), 297 and 311.

47 One *ḳıyye* was equal to 1 282 grams.

48 BNA, ‘Half a Dozen Leeches’, *op. cit.* (note 21).

49

Mutlu Özgen , ‘Tokat Kentinin Ticaret Mekanları’, Vakıflar Dergisi, 31 (2008), 172, 173.

50 BOA (Prime Ministry Ottoman Archives), I.MVL 214/7082, 1 Ramadan 1267 [30 June 1851].

51 BOA, MB.I. 12/90, 23 Jumada al-Thani 1270 [23 March 1854].

52 Sawyer, ‘The Portuguese Leech Trade’, *op. cit.* (note 33), 288.

53 BOA, I.MVL 214/7082, 1 Ramadan 1267. Forty *para* were equal to one *gurush.*

54 Ardeleanu, *op. cit.* (note 3), 187.

55

Karen Barkey, The Empire of Difference: the Ottomans in Comparative Perspective (New York: Colombia University Press, 2008), 230 .

56

Huricihan İslamoğlu , Osmanlı İmparatorluğu’nda Devlet ve Köylü (Istanbul: İletişim Yayınları, 2018), 27 .

57

Nadir Özbek , ‘Tax Farming in the Nineteenth-Century Ottoman Empire: institutional Backwardness or the Emergence of Modern Public Finance?’, Journal of Interdisciplinary History, XLIX, 2 (Autumn, 2018), 235 .

58 BOA, HAT 282/16777, 29 Dhu al-Hijjah 1252 [6 April 1837].

59 BOA, HAT 530/ 26162, 29 Dhu al-Hijjah 1252 [6 April 1837].

60 BOA, I.MVL. 1/3, 11 Muharram 1256 [15 March 1840].

61 BOA, I.DH. 7/340, 26 Dhu al-Hijjah 1255 [1 March 1840].

62 BOA, A.MKT. 37/27, 8 Rabi al-Awwal 1262 [6 March 1846].

63 BOA, A.MKT. 53/1, 3 Dhu al-Qadah 1262 [23 October 1846].

64
*Ibid.*

65 BOA, A.MKT. 52/83, 1 Dhu al-Qadah 1262 [21 October 1846].

66 BOA, ŞD. 2115/44, 22 Jumada al-Thani 1287 [11 June 1871].

67 BOA, C.ML. 111/4926, 20 Shaban 1252 [30 November 1836].

68 BOA, HR.SYS. 1338/75, 14 Rabi al-Thani 1275 [21 November 1858].

69 BOA, A.MKT.NZD. 68/46, 25 Safar 1269 [8 January 1852].

70 BOA, C.ML. 91/4109, 5 Safar 1269 [18 November 1852].

71 BOA, HR.MKT. 654/25, 22 Safar 1286 [3 June 1869].

72 BOA, C.ML. 91/4109, 5 Safar 1269 [18 November 1852].

73 BOA, C.AS. 244/10275, 17 Shawwal 1255 [24 December 1839].

74 BOA, HR.MKT. 54/21, 26 Rabi al-Awwal 1269 [7 January 1853].

75 BOA, C.SH. 24/1191, 29 Rabi al-Awwal 1260 [18 April 1844].

76 Fatih Gencer, ‘Hünkâr İskelesi Antlaşması’nın Osmanlı-İngiliz İlişkilerine Yansımaları’, *Tarih Araştırmaları Dergisi, 34/58* (2015), 629–650.

77 Özbek, *op. cit.* (note 57), 226.

78

William D. Grampp , ‘How Britain Turned to Free Trade’, Business History Review, 61, 1 (1987), 87.

79

Frank Edgar Bailey , British Policy and The Turkish Reform Movement: a Study in Anglo-Turkish Relations 1826-1853 (Cambridge: Harvard University Press, 1942), 126.

80 Viscount Palmerston, Commons Sitting, 11 February 1839, https://api.parliament.uk/historic_hansard/commons/1839/feb/11/treaty-of-commerce-with-austria, (accessed 4 June 2021).

81 Bailey, *op. cit.* (note 79), 128.

82

Şevket Pamuk, Uneven Centuries: economic Development of Turkey Since 1820 (Princeton/Oxford: Princeton University Press, 2018), 98 .

83 Cited from Lord Palmerston’s letter of 6 February 1838, by Bailey, *op. cit.* (note 79), 124.

84

Şevket Pamuk , Osmanlı-Türkiye İktisadi Tarihi 1500-1914 (Istanbul: İletişim Yayınları, 2003), 211.

85

Mehmet Genç , ‘19. Yüzyılda Osmanlı İktisadî Dünya Görüşünün Klasik Prensiplerinde Değişmeler’, in Devlet ve Ekonomi (Istanbul: Ötüken Yayınları, 2013), 83–92 .

86 BOA, MHD. 56/03, 5 Jumada al-Thani 1255 [16 August 1839].

87 Although the system has been translated as monopoly, there is a dispute over the meaning of it in the archival documents. Mübahat Kütükoğlu, ‘Baltalimanı Muahedesi’, *Türkiye Diyanet Vakfı İslam Ansiklopedisi*, 5 (Istanbul, 1992), 38–40.

88
*Ibid.*

89 Mehmet Genç, ‘Yed-i Vahid’, *Türkiye Diyanet Vakfı İslam Ansiklopedisi,* 43 (Istanbul, 2013), 378–383.

90 Pamuk, *Uneven Centuries*, *op. cit.* (note 82), 97.

91 BOA, I.MVL. 1/3, 11 Muharram 1256 [15 March 1840].

92
*Ibid.*

93

Stanley Lane-Poole , The Life of the Right Honourable Stratford Canning II (London: Longmans, Green, and Co., 1888), 56 .

94 NA (The British National Archives), FO 424/13/61, from Stratford Canning to Earl of Aberdeen, 1 April 1844.

95 NA, FO 424/24/166, from Stratford Canning to Earl of Aberdeen, 2 August 1844.

96

Mübahat Kütükoğlu , Osmanlı-İngiliz İktisâdi Münasebetleri II (1838-1850), (Istanbul: Edebiyat Fakültesi Basımevi, 1976), 50 .

97 A tube takes approximately 3 000 leeches, ‘A Chapter About Leeches’, *Bell’s Weekly Messenger*, 19 January 1852.

98 BOA, A.MKT. 213/42, 23 Shaban 1265 [14 July 1849].

99 BOA, HR.TO. 212/32, 16 Shaban 1265 [7 July 1849].

100 BOA, HR.TO. 199/83, 7 Shaban 1278 [7 February 1862].

101 Kütükoğlu, *Osmanlı-İngiliz İktisâdi Münasebetleri, op. cit.* (note 95), 47.

102 NA, FO 424/13/61, from Stratford Canning to Earl of Aberdeen, 1 April 1844.

103 BNA, *Morning Advertiser*, 20 January 1845.

104 Kütükoğlu, ‘Baltalimanı Muahedesi’, *op. cit.* (note 87).

105 NA, FO. 424/2/1, from J. Dodson to Sir Stratford Canning, 31 May 1843.

106 BNA, *Newcastle Courant*, 28 December 1838; *Globe*, 8 June 1839; BOA, Y.EE. 91/5, H. 07. 06. 1254.

107 NA, FO. 424/7/65, from the Earl of Aberdeen to Lord Stuart de Rothsay, 26 December 1843; FO. 424/9/39, from the Earl of Aberdeen to Sir Stratford Canning, 19 March 1844.

108 NA, FO. 424/9, from Mr. Mac Gregor to Mr. Addington, 20 December 1843.

109 BNA, *Morning Advertiser*, 15 December 1845.

110 Kütükoğlu, ‘Baltalimanı Muahedesi’, *op. cit.* (note 87).

111
*Ibid.*

112

Ronald Findlay and Kevin H. O’Rourke , ‘Commodity Market Integration, 1500-2000’, Globalization in Historical Perspective (University of Chicago Press, 2003), 37 .

113 Mübahat Kütükoğlu, ‘Tanzimat Devri Osmanlı-İngiliz Gümrük Târifeleri’, *İstanbul Üniversitesi Edebiyat Fakültesi Tarih Enstitüsü Dergisi,* 4–5 (1973–1974), 338 and 342.

114
*Ibid.*, 350.

115 Ardeleanu, *op. cit.* (note 3), 179.

116 Freeman Hunt, ‘Trade of the Ottoman Empire’, *Hunt’s The Merchant’s Magazine and Commercial Review,* 30 (April, 1854), 413.

117 Idiom of ‘capitalist leech’ became an expression showing the dark side of capitalism. Robert G.W. Kirk and Neil Pemberton , Leech (London: Reaktion Books, 2013), 75 .

118

William Cronon , Changes in the Land: indians, Colonists, and the Ecology of New England (New York: Hill and Wang, 2003), 98 .

119
*Ibid.*, 98.

120

Dale F. Lott , American Bison: a Natural History (Berkeley/London: University of California Press, 2002), 167 .

121

John R. McNeill , Something New Under the Sun: an Environmental History of the Twentieth-Century World (New York: W. W. Norton Company, 2000), 320 .

122
*Ibid.*, 319.

123 Ardeleanu, *op. cit.* (note 3), 177.

124
*Ibid.*, 181–184.

125 Sawyer, ‘The Portuguese Leech Trade’, *op. cit.* (note 33), 311.

126
*Ibid.*, 308.

127 Ardeleanu, *op. cit.* (note 3), 184.

128 BOA, C.ML. 91/4109, 5 Safar 1269 [18 November 1852].

129 BOA, MVL. 906/38, 23 Jumada al-Awwal 1276 [18 December 1859].

130 BNA, *Morning Advertiser*, 20 January 1845.

131 BOA, HR.TO. 212/36, 26 Shaban 1265 [17 July 1849].

132 BOA, HR.TO. 219/52, 25 Dhu al-Qadah 1269 [30 August 1853].

133 There is another stamp of a consulate which is not clear. BOA, MVL. 906/38, 21 Jumada al-Awwal 1276 [16 December 1859].

134 BOA, HR.MKT. 54/21, 26 Rabi al-Awwal 1269 [7 January 1853].

135 It is not clear from the archival sources when this committee was established. BOA, HR.TO. 195/19, 30 Jumada al-Awwal 1270 [28 February 1854].

136 BOA, A.AMD. 59/89, 29 Dhu al-Hijjah 1271 [12 September 1855].

137
*Ibid.*, 316.

138 Sawyer, ‘The Portuguese Leech Trade’, *op. cit.* (note 33), 295.

139 Ardeleanu, *op. cit.* (note 3), 182 and 185.

140 BOA, A.MKT.UM. 4/68, 15 Safar 1266 [31 December 1849].

141 BOA, C.ML. 91/4109, 5 Safar 1269 [18 November 1852]. Also, see Table 4 for Rusuvich’s calculations.

142 BOA, HR.TO 417/19, 21 Jumada al-Awwal 1268 [13 March 1852]. He also reported export rates of certain harbours within the Empire (see Table 2).

143 BOA, C.SH. 26/1280, 4 Safar 1264 [11 January 1848].

144 BOA, I.MVL. 214/7082, 1 Ramadan 1267 [30 June 1851].

145 BOA, HR.MKT. 54/21, 26 Rabi al-Awwal 1269 [7 January 1853].

146
*Ibid.*

147 BOA, I.ŞD. 26/1174, 25 Dhu al-Hijjah 1289 [23 February 1873].

148 BOA, A.MKT.MHM. 35/53, 14 Shawwal 1267 [12 August 1851].

149 BOA, A.MKT.DV. 141/29, 28 Muharram 1276 [27 August 1859]. These examples also show the amount of taxes annually.

150 BOA, I.MVL. 582/26153, 11 Shaban 1284 [8 December 1867].

151 BOA, I.MVL. 472/21381, 29 Safar 1279 [26 August 1862].

152 Özlem Sert, ‘Water, Firewood, and Disease in Nineteenth-Century Istanbul’, Environment and Society Portal, *Arcadia,* 45 (Autumn 2020), Rachel Carson Center for Environment and Society.

153 Alfredo Morabia, ‘P.C.A. Louis and the Birth of Clinical Epidemiology’, *J. Clin. Epidemiol.,* 49, 12 (1996), 1327–1333.

154 Magner, *op. cit.* (note 12), 507 and 530.

155
*Ibid*, 256.

156 For detailed information, see the websites, *International Community of Hirudopractitioners,* https://hiruline.com/en/international_community/ and *International Leech Organisation*, http://www.leech.ru/en/.

157
*Trade in Endangered Species Act 1989*, 129, https://cites.org/, (accessed 11 December 2021). Also, the British government started a recovery project for the medicinal leeches, accessed 4 September 2022, https://freshwaterhabitats.org.uk/projects/medicinal-leech-recovery-project/.

